# Pediatric paraganglioma of the posterior mediastinum: A case report and review of literature

**DOI:** 10.1097/MD.0000000000011212

**Published:** 2018-07-06

**Authors:** Miao Yuan, Chang Xu, Gang Yang, Weiya Wang

**Affiliations:** a Department of Pediatric Surgery; b Department of Pathology, West China Hospital, Sichuan University, Chengdu, Sichuan, P.R. China.

**Keywords:** children, paraganglioma, posterior mediastinum

## Abstract

**Rationale:**

Paraganglioma is rare in children and most pheochromocytomas originate in the adrenal gland.

**Patient concerns:**

The clinical characteristics, diagnosis, and managements in a 9-year-old boy who presented with vomiting and occasional headache with a blood pressure of 210/170 mm Hg was retrospectively reviewed. CT scan of the chest revealed a 7 × 5-cm-sized soft tissue mass in the left paraspinal area. Biochemical reports revealed elevated levels of serum norepinephrine, urine norepinephrine, urine dopamine, and serum neuron specific enolase.

**Diagnoses:**

The immunohistochemical studies suggested that the tumor was a paraganglioma of the posterior mediastinum.

**Interventions:**

The patient underwent mass resection with thoracotomy. Before operation, the patient was prepared by orally administering captopril, propranolol hydrochloride, and phenoxybenzamine. Body fluid volume was also prepared by vein and mouth in 3 days.

**Outcomes:**

The patient was followed up postoperatively for 1 year without recurrence.

**Lessons:**

We should be highly vigilant the pediatric tumor of the posterior mediastinum with vomiting and headache as the first clinical manifestation.

## 1 Introduction

Pheochromocytomas and paragangliomas, which arise from chromaffin cells of the adrenal medulla and extra-adrenal paraganglia, respectively, are uncommon. Approximately 95% of catecholamine-secreting tumors are located in the abdomen and pelvis.^[1]^ Pediatric paraganglioma of the posterior mediastinum is reported to be exceedingly rare, and there is a lack of guidelines for its perioperative managements. Here, we report a rare case of a paraganglioma of the posterior mediastinum in a 9-year-old boy.

## 2 Consent

The patient's parents provided informed consent for the publication of his clinical and radiological data. This case report was approved by Medical Ethical Committee of West China Hospital, Sichuan University.

## 3 Case report

**Figure 1:**
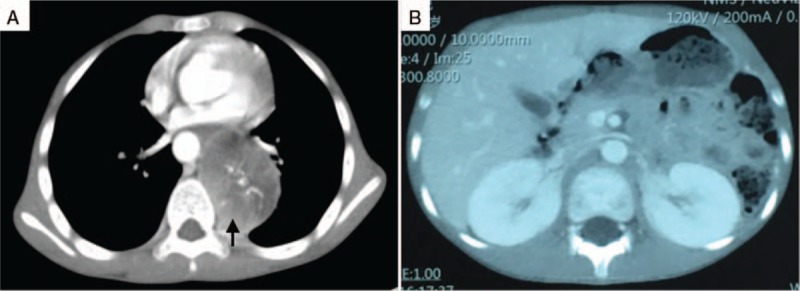
A, CT scan of the chest showed a 7 × 5-cm-sized soft tissue mass on the left paraspinal with destroying adjacent thoracic vertebra and ribs (arrow, the tumor). B, CT scan of the abdomen showed normal. CT = computed tomography.

A 9-year-old boy presented to a local hospital with vomiting and occasional headache with a blood pressure of 210/170 mm Hg. No obvious diseases were observed on digestive endoscopy and abdominal computed tomography (CT) scan, and no remarkable improvement by medicine treatment. CT scan of the chest revealed a 7 × 5-cm-sized soft tissue mass in the left paraspinal area from T3 to T7 with destruction of the adjacent thoracic vertebra and ribs (Fig. 1). Biochemical reports revealed elevated levels of serum norepinephrine, urine norepinephrine, urine dopamine, and serum neuron specific enolase. Serum epinephrine, urine epinephrine, alpha fetoprotein, and carcinoembryonic antigen were within the normal range (Table 1). The admitting diagnosis was tumor in the posterior mediastinum: paraganglioma? Before operation, the patient was prepared by orally administering captopril, propranolol hydrochloride, and phenoxybenzamine by mouth. The patient's blood pressure remained stable at approximately 110/80 mm Hg. In addition, body fluid volume was also prepared by vein and mouth in 3 days before surgery.

**Table 1:**
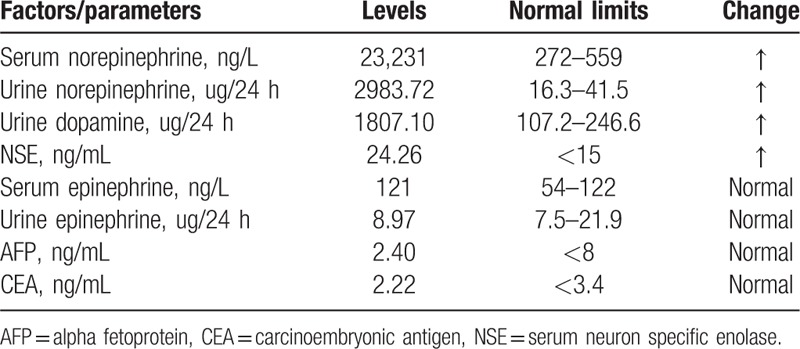
Levels of biochemical test.

**Figure 2:**
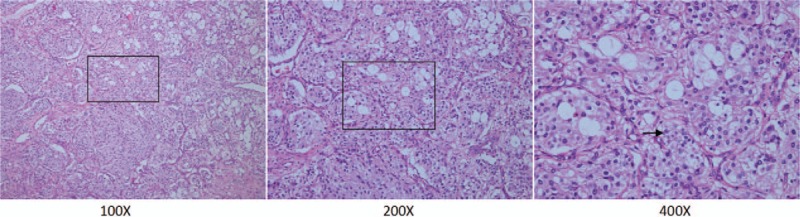
Histologic studies showed eosinophilic granular cytoplasm with hyperchromatic and round nuclei (arrow, hematoxylin and eosin, magnification ×100, ×200, ×400).

**Figure 3:**
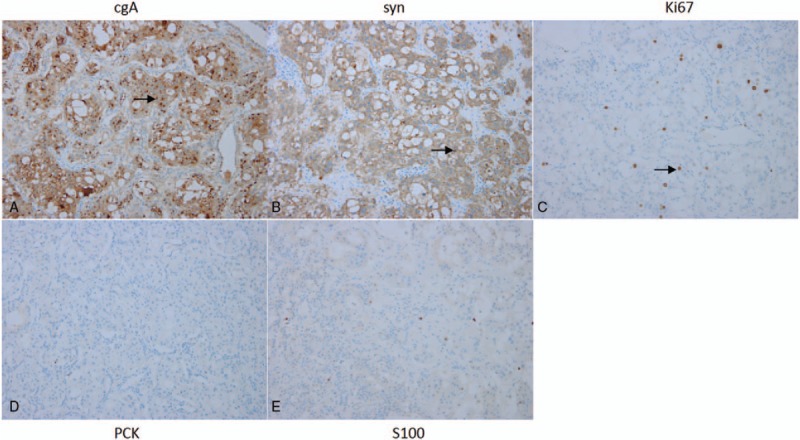
cgA and syn antibody IHC staining showed positive reactivity (A, B, arrow). The positive rate of Ki67 staining was 2% to 5% (arrow); S100 and PCK antibody IHC staining showed negative reactivity (D, E); magnification ×200. IHC = immunohistochemical.

**Figure 4:**
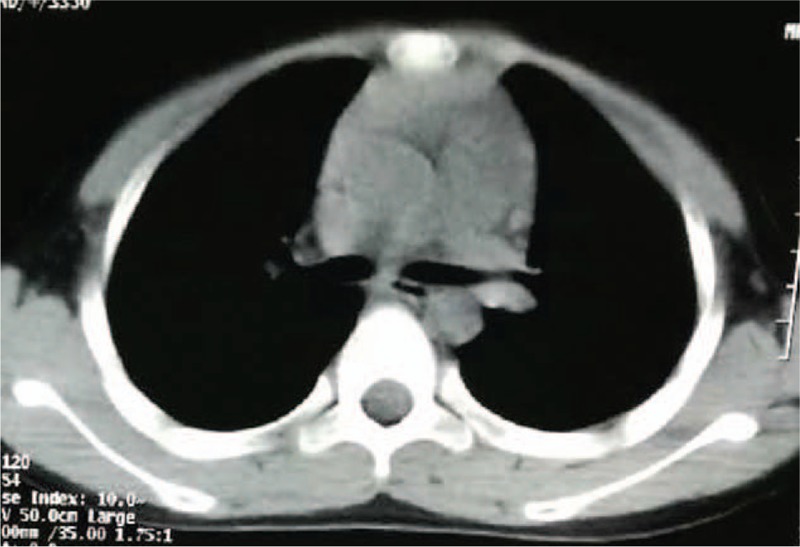
Postoperative CT scan of the chest showed negative for tumor recurrence.

Thoracotomy was performed through the left fifth intercostal space. Intraoperatively, several membranous and fascicular adhesions existed in the thoracic cavity. The irregular ovoid mass measured 8 × 7 × 5 cm. The tumor originated from the nerve root and adhered to the surrounding tissue. It invaded the spine and chest wall. The mass was tough and rich in blood supply. There were intraoperative changes in the patient's blood pressure, which ranged from 85/50 mm Hg to 180/130 mm Hg. During the resection, the surgeon closely communicated with the anesthesiologist to decide the operative process. Histological studies demonstrated that the mass was a tumor (Fig. 2). Immunohistochemical (IHC) studies demonstrated that tumor cells stained positive for synaptophysin (syn, +) and chromogranin A (cgA, +). The positive rate of Ki67 (MIB-1) staining was 2% to 5%. The S100 and PCK staining was negative (Fig. 3). The immunohistochemical studies suggested that the tumor was a paraganglioma. Postoperatively, the patient's blood pressure was stable and within the normal range. On postoperative day 2, the concentration of serum epinephrine was 236 ng/L and serum norepinephrine was 4686 ng/L. On postoperative day 4, serum norepinephrine (321 ng/L) and epinephrine (76 ng/L) were normal. The patient was discharged on postoperative day 6. After operation, the patient did not exhibit hypertension and his blood pressure was normal without medicine. After 1 year, follow-up chest CT did not reveal tumor recurrence (Fig. 4).

## 4 Discussion

Patients with paragangliomas often present with symptoms secondary to catecholamine excess. The common symptoms are headache, palpitations, sweating, nausea, vomiting, and weight loss. Hypertension is observed in 70% to 90% of children with pheochromocytoma.^[2]^ Except the presenting symptoms, the catecholamines in the blood and metabolites in the urine are always elevated.^[3]^ Radiological tests are helpful to determine the tumor location. It is important to distinguish from the other tumors before operation. Furthermore, α-adrenergic or/and β-adrenergic blockade, which are considered to decrease the risk of cardiac dysrhythmia accompanied with pheochromocytoma during the surgery, are used to maintain normal blood pressure normal.^[4,5]^ Meanwhile, patients with pheochromocytoma usually have low blood volume^[6]^ and it is necessary to counteract hypovolemia after removing the tumor due to sudden decrease in serum catecholamine levels.^[7]^ Our patient presented the same symptoms such as vomiting, headache, and hypertension, as those previously reported in the literature. Preoperative biochemical studies revealed that serum and urine norepinephrine were elevated and serum and urine epinephrine were within the normal range. The results suggested that the tumor mainly produced norepinephrine. Postoperatively, serum norepinephrine continued to decline. However, serum epinephrine increased on postoperative day 2 and then was within the normal range, which may be due to the intraoperative extrusion of the tumor. To maintain stable intraoperative blood pressure, blood volume was prepared for physiological need of sodium solution intravenously and orally on preoperative day 3.

Renard et al^[8]^ reviewed the surgery and perioperative care required for patients with pheochromocytoma and paraganglioma. Surgical resection for paraganglioma was regarded as the gold standard of treatment options. Intravenous midazolam and propofol were used as anesthesia. Tsirlin et al^[1]^ suggested that fentanyl, ketamine, and morphine should be avoided because they may stimulate catecholamine release. Intraoperatively, tumor vessels were first managed to prevent catecholamine entering the vascular structure. Meanwhile, it was necessary to closely communicate with the anesthesiologist to control hemodynamics. Once the blood pressure was elevated, the operation was suspended. Vasopressin was used for hemodynamic resuscitation to control acute catecholamine deficiency after tumor removal.^[9]^

The tumor was excised for histological and immunohistochemical examinations. The accurate diagnosis of paraganglioma could be based on immunohistochemical staining of syn, cgA, Ki-67, S100, and PCK. However, the definition of malignancy in paraganglioma was based on not only histological and immunohistochemical characteristics but also the presence of metastasis and the size of the tumor.^[10]^ Owing to the relative rarity of the tumor, chemotherapy for children was absent and unsatisfactory.^[11]^ In our patient, preoperative CT of the chest and abdomen suggested no other problems besides the mass in the posterior mediastinum. Meanwhile, surgical resection was complete. Postoperatively, the symptoms of vomiting and headache disappeared. The patient's blood pressure remained normal without the use of hypotensors. Biochemical test results revealed that serum and urine norepinephrine and epinephrine levels returned to normal. Our patient decided to undergo long-time follow-up without chemotherapy. During the follow-up, monitoring the patient's symptoms and blood pressure was important. CT of the chest and biochemical studies are essential 3 months, 1 year, and then each year postoperatively.

## 5 Conclusions

The experience of treating pediatric paraganglioma of the posterior mediastinum is insufficient. Our treatment was based on some case reports and experience with adults. Our patient was rehabilitated and discharged using a series of perioperative managements. However, pathological diagnosis could not provide benign or malignant diagnosis, and long-term follow-up is still needed.

## Author contributions

**Conceptualization:** Chang Xu.

**Data curation:** Gang Yang, Weiya Wang.

**Formal analysis:** Gang Yang.

**Writing – original draft:** Miao Yuan.

**Writing – review & editing:** Chang Xu, Weiya Wang.

## References

[1] Tsirlin A Oo Y Sharma R Pheochromocytoma: a review. Maturitas 2014;77:229–38. 24472290 10.1016/j.maturitas.2013.12.009

[2] Caty MG Coran AG Geagen M Current diagnosis and treatment of pheochromocytoma in children. Arch Surg 1990;125:978–81. 2378563 10.1001/archsurg.1990.01410200036004

[3] Spector JA Willis DN Ginsburg HB Paraganglioma (pheochromocytoma) of the posterior mediastinum: a case report and review of the literature. J Pediatr Surg 2003;38:1114–6. 12861555 10.1016/s0022-3468(03)00208-2

[4] Ellis D Gartner JC The intraoperative medical management of childhood pheochromocytoma. J Pediatr Surg 1980;15:655–9. 6108356 10.1016/s0022-3468(80)80519-7

[5] O’Neill J James A Rowe MD Pediatric Surgery. St Louis, MO: Mosby; 1998.

[6] Deoreo GA Jr Stewart BH Tarazi RC Preoperative blood transfusion in the safe surgical management of pheochromocytoma: a review of 46 cases. J Urol 1974;111:715–21. 4151537 10.1016/s0022-5347(17)60053-3

[7] Iijima T Iwao Y Ito Y Perioperative circulating blood volume analysis in management of a 13-year-old female patient with an extraadrenal pheochromocytoma and refractory ventricular tachycardia: a case report. J Pediatr Surg 2006;41:e15–7. 10.1016/j.jpedsurg.2006.04.007 16863830

[8] Renard J Clerici T Licker M Pheochromocytoma and abdominal paraganglioma. J Visc Surg 2011;148:e409–16. 21862435 10.1016/j.jviscsurg.2011.07.003

[9] Lord MS Augoustides JG Perioperative management of pheochromocytoma: focus on magnesium, clevidipine, and vasopressin. J Cardiothorac Vasc Anesth 2012;26:526–31. 22361482 10.1053/j.jvca.2012.01.002

[10] Holwitt D Neifeld J Massey G Case report of an 11-year-old child with a nonfunctional malignant pheochromocytoma. J Pediatr Surg 2007;42:E13–5. 10.1016/j.jpedsurg.2007.08.046 18022420

[11] Ross JH Pheochromocytoma: special considerations in children. Urol Clin North Am 2000;27:393–402. 10985140 10.1016/s0094-0143(05)70088-4

